# Student acceptance and evolving teacher roles in mixed reality dance education

**DOI:** 10.1038/s41598-025-27493-w

**Published:** 2025-11-23

**Authors:** Yang Meng, Ng Khar Thoe, Abd Razak Nurul Nadiah

**Affiliations:** 1 Shaanxi University of International Trade & Commerce, Xianyang, China; 2 UCSI University, Kuala Lumpur, Malaysia; 3 INTI International University, Nilai, Malaysia; 4 University of Malaya, Kuala Lumpur, Malaysia

**Keywords:** Mixed reality (MR), Higher education, Personalized learning, Emerging technologies, Sustainable development education, Education, Information systems and information technology, Psychology, Psychology

## Abstract

With the ongoing digitalization of dance education, there remains a lack of instruments to evaluate students’ needs concerning the integration of Mixed Reality (MR). This study aimed to develop the MR Dance Attitude Scale (MR-DAS) based on Gardner’s Multiple Intelligence (MI) Theory as the conception framework and to investigate how MR acceptance may reshape future teaching roles. A mixed-methods approach was employed, comprising a validated questionnaire administered to 110 dance majors and semi-structured interviews (*n* = 7). Validity and reliability were assessed using SPSS 26.0. Quantitative results demonstrated strong suitability for factor analysis (KMO = 0.926, *p* < 0.001). Exploratory Factor Analysis extracted 27 items grouped into three dimensions: Learning Impact, MR Teaching Intention, and Equipment Requirements. Qualitative findings highlighted multiple barriers influencing technology acceptance. Findings indicated that students are highly receptive to MR due to its potential to enhance learning efficiency and interactivity; however, they also emphasized the irreplaceable role of human teachers in fostering creativity, interpreting culture, and ensuring safety. These insights support a collaborative model combining teachers and MR technologies. The validated instrument as a reliable assessment instrument, is capable of identifying the varied learning needs of students, and offers a practical resource for educators aiming to further investigate the application and advancement of MR technology in educational contexts. This contributes to the realization of sustainable and quality education aligned with Sustainable Development Goal 4.

## Introduction

 Dance education has entered the era of digital transformation characterized by web 3.0. An increasing number of innovative integrations of emerging technologies, such as Virtual Reality (VR) and Augmented Reality (AR), with educational practices are being observed in teaching contexts. However, the absence of a reliable and valid instrument to systematically measure student attitudes and intentions toward MR-integrated dance education remains a significant gap in the literature. This gap impedes empirical research and hinders the innovative implementation of emerging technologies within the dance curriculum. After conducting research, the author found that there are currently no validated instruments and theory available for investigating student intentions regarding the use of Mixed Reality (MR) technology to study dance. To address this gap, the author employed the Theory of Multiple Intelligence proposed by Harvard psychologist Howard Gardner^1^, as the conceptual framework for designing and developing the measurement scale items. Aimed to develop and validate a new instrument specifically designed for the college students. Based on the journals submitted by students during the research period, online dance lessons identify, understand, and systematically summarize their needs. Then the author developed and validated the Mixed Reality Dance Attitude Scale (MR-DAS), specifically designed to survey college students’ perceptions of intentions and attitudes regarding the use of MR technology in dance class.

### Concept framework of MR-DAS

**Fig. 1:**
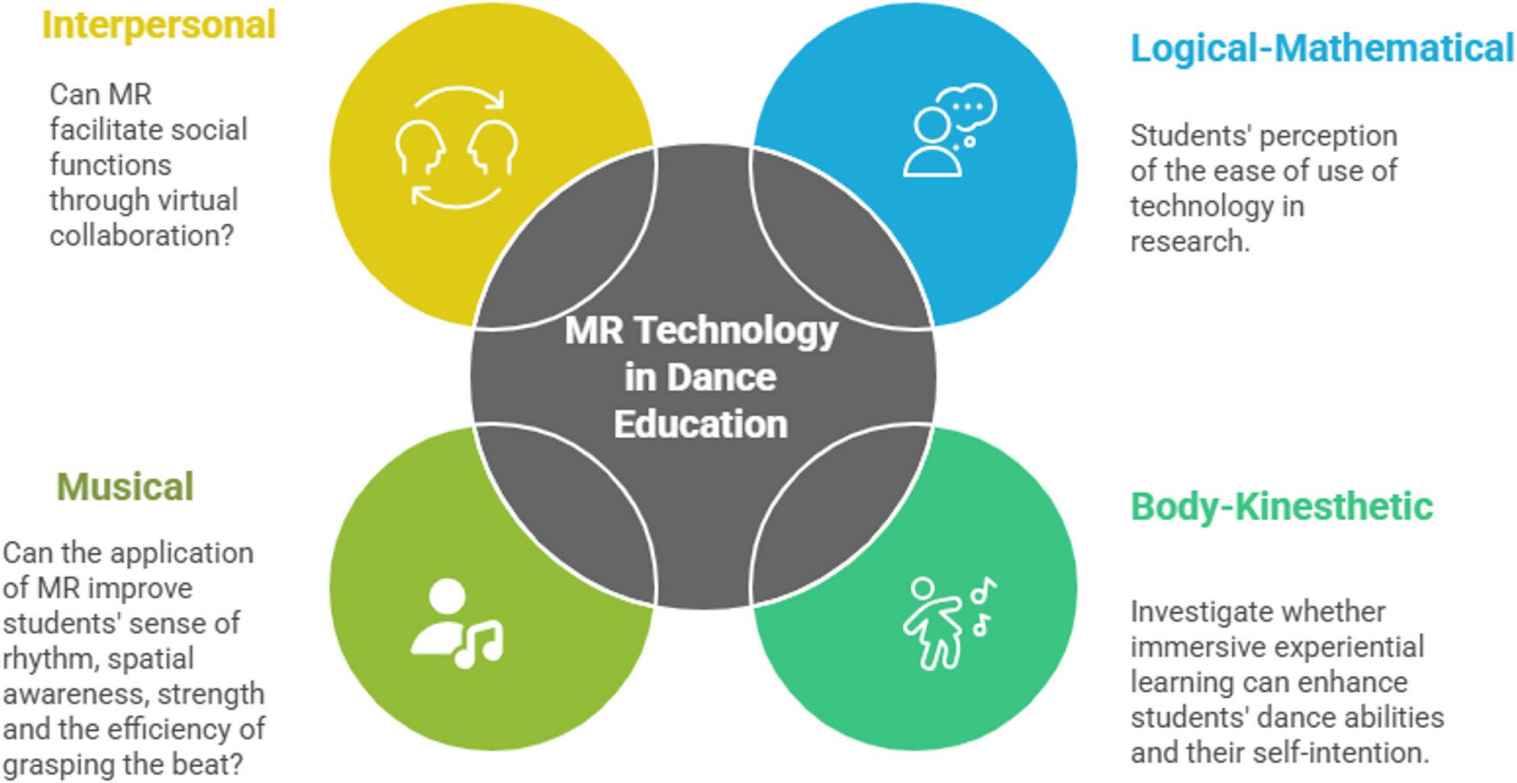
Based on the four measurement indicators and application descriptions within Gardener’s MI conceptual framework.

Mixed reality (MR) technology, which enables holographic guidance, spatial annotation, and real-time motion correction in three-dimensional space^2^, is progressively emerging as a transformative teaching approach within the realm of dance education. While its efficacy has been substantiated in surgical training^3^ and sports training^4^, rigorous validation of MR technology specifically in dance education remains underdeveloped. Notably, there is currently a paucity of standardized assessment tools capable of effectively evaluating the diverse metrics associated with MR applications in dance pedagogy. To address this gap, this study employs the Theory of Multiple Intelligence, proposed by Harvard psychologist Howard Gardner^1^, as the conceptual framework for designing and developing the measurement scale items. The author from nine types of human intelligence:1. Visual-spatial, 2. Linguistic-verbal, 3. Logical-mathematical, 4. Body-kinesthetic, 5. Musical, 6. Interpersonal, 7. Intrapersonal, 8. Naturalistic, 9. Existential intelligence, four indicators were selected for this study to validate the development of the research tool regarding students’ intention to integrate MR technology into dance classes as follows in Fig. 1.

### The application of diverse ICT in dance education

While teaching platforms or technology instruments have become increasingly standardized and widely adopted, the author argues that the application of Information and Communication Technology (ICT) during this period primarily served to enhance the engagement and interest of learners. However, studies consistently point out that the use of these platforms in online education affects the interaction between teachers and students, and there are also problems with delayed feedback, and students will face great challenges in kinesthetic learning in this online classroom environment^5–7^. Recent studies have indicated that several scholars have proposed the integration of emerging technologies into educational contexts. For instance, although platforms like YouTube and Zoom provide convenient learning opportunities^6,8^, the Zuvio IRS platform was also introduced by some scholars at the onset of the pandemic^9^. During the peak of the pandemic in 2021, educators turned to blended online teaching models such as MOOC + SPOC (Open edX) for dance instruction^10^. Additionally, educators experimented with various tools, including MOOC videos, dance tutorials, video MVs, WeChat, and QQ, as means of facilitating online education^11^. By 2021, several authors noted that Zoom, Cisco, Webex, Google Class, Panopto^12^ had emerged as one of the most popular tools during that time. The diversified development of teaching platforms has been accelerated. Researchers have discussed in their articles how the use of new technologies can stimulate students’ interest in learning, What results did it bring^10,12,13^. Zoom, Cisco Webex, Panopto, Canvas, Webinar, (LMS), and online exams. Systems, e-books, online tutorials, simulation experiment software, VR, AR, EXD and other interesting emerging technologies are beginning to be integrated with dance education.

### Existing scales to measure new emerging technology attitudes

As for researchers, Artificial intelligence-based posture assessment for self-evaluation has been suggested^6^, existing^14^, along with an e-learning acceptance framework grounded in the Unified Theory of Acceptance and Use of Technology (UTAUT) model^14,15^. Additionally, a multi-model toolkit designed for visually impaired learners has been developed^16^. Nevertheless, these approaches remain constrained by limitations in teaching tools or reliance on learner motivation, thereby making it challenging to replicate specialized dance instruction methods.

For instance, Coelho and Menon^15^ investigated the adoption of online dance learning. However, their research mainly focuses on traditional online learning and video teaching^17^, while ignoring the immersive teaching environment that MR technology can bring to people. Similarly, the dance formative assessment framework proposed by Ling and Yang^18^ primarily emphasizes on-screen recorded performances but remains constrained within the traditional two-dimensional spatial research paradigm^18^. These cases have also failed to adequately account for the real-time auxiliary assessment capabilities of MR technology. Consequently, the author posits that MR, as a technical approach for dance education, still holds potential for further development.

### The present study and research questions

To enhance the integration of MR technology with dance education, the author developed and validated the Mixed Reality Dance Attitude Scale (MR-DAS). The measurement tool developed by the author based on the theory of Multiple Intelligence (MI) is specifically designed to assess several key needs of students majoring in dance.

- The impact of the integration of emerging technologies on learning itself.
- College students’ acceptance of the integration of emerging technologies into MR dance teaching lessons.
- The correlation between MR technology and equipment support for students’ learning.

And the correlation between the various variables in the scale.

The author conducted a questionnaire survey among 110 college students who had participated in school-based dance classes, and further evaluated the reliability, structural validity, and correlation analysis of the Mixed Reality Dance Attitude Scale (MR-DAS). This study provides a foundational tool for educators to adopt future immersive dance teaching methods and integrate emerging technologies into dance education research. This paper addresses the following three research questions (RQs) have been formulated.

RQ1: Is “MR-DAS” a reliable measurement tool?

RQ2: Is “MR-DAS” an effective measurement tool?

RQ3: Is there any evidence that the MR dance education promotes development of multiple intelligence (MI) based on the response of students participating in this study?

RQ4: What key areas that learners’ needs should be emphasized in MR dance education moving forward? And explore the implications of these demands for the future positioning of the role of teachers in dance education.

## Materials and methods

The data collection procedures for this study were reviewed and approved by the Ethics Review Committee (Approval Number: IEC-2025-FOSSLA-0032). All participants provided written informed consent prior to their inclusion in the study, having been fully informed of the study’s purpose, procedures, and measures for privacy protection. The study was conducted in full compliance with the ethical principles outlined in the Declaration of Helsinki, as well as the relevant research regulations of China/Shaanxi. All research activities adhered to internationally recognized standards of scientific ethics.

### Development of survey questionnaire

The research questionnaire consists of 29 items. This questionnaire is part of the author’s doctoral dissertation scale. The survey covers student demographics, general intentions, teaching methods, awareness and attitude towards the use of MR technology in dance lessons, and the extent of demand for equipment utilized to implement this technology. The tool was constructed after joint content verification by two experts from the fields of artificial intelligence and pedagogy. The initial version of the questionnaire was validated by two designated experts (an education doctoral supervisor and an artificial intelligence specialist) through telephone interviews and content validation procedures. Prior to the pilot test involving 10 randomly selected university students, the questionnaire’s readability and validity were assessed to ensure its clarity and acceptability. Subsequently, all participants’ feedback was integrated, leading to a revised version with enhanced comprehensibility and a more logical question sequence. Finally, the definitive questionnaire was distributed online to students within the university where the author is employed.

### Data collection and validation process

The questionnaire was validated by an interdisciplinary team comprising senior researchers (experts) in the fields of education and artificial intelligence, as well as university faculty members (author). The experts involved in this process rigorously evaluated the completeness of the questionnaire and identified potential ambiguities or misunderstandings within its content. Special attention was given to the structural design of the questionnaire to ensure it can be clarity and comprehensibility. To ensure reliability and validity, a pilot study involving 50 students was conducted before the formal data collection. In the subsequent formal investigation we distributed 110 questionnaires. Participants were able to access the Wenjuan Star platform via provided links or QR codes to complete the questionnaire as part of this investigation.

After content validation, the authors conducted data collection via an online survey from April 10 to April 17. The participants were undergraduate students involved in a research study organized by Shaanxi University of International Trade & Commerce. All participants were completely informed about the study’s details and voluntarily confirmed their consent to participate. Personal information was not recorded, and all questionnaires were completed anonymously. Written approval for the study was obtained from Shaanxi Institute of International Trade & Commerce.

### Statistical analysis

 Prior to conducting the statistical analysis, the collected questionnaires were systematically reviewed for completeness and data quality. All 110 returned questionnaires were completely completed with no missing responses, thereby eliminating the need for data imputation or participant exclusion. The dataset was subsequently imported into SPSS for additional analysis. To establish the construct validity of the questionnaire, exploratory factor analysis (EFA) was conducted to uncover the underlying factor structure. Exploratory factor analysis (EFA) was conducted using principal component analysis (PCA). Varimax rotation is appropriate for datasets characterized by independent factors^19^. However, given the anticipated intercorrelations among factors in this study, given the anticipated interrelationships among theoretical constructs (e.g., learning impact and teaching intention), an oblique rotation method was considered appropriate. Therefore, we considered that the Promax rotation was employed during the EFA to better capture the underlying complex and interrelated factor structure^20–23^. Promax rotation was applied with a kappa value of 4 to facilitate the extraction of interpretable factors. Accordingly, the level for acceptable factor loading was set at >0.5, while eigenvalues exceeding 1 were retained. The Kaiser–Meyer–Olkin test was also used to measure the adequacy of the sample size for EFA, with values >0.7 considered as acceptable. Additionally, Bartlett’s test of sphericity assessed the suitability of the correlation matrix for factor analysis, yielding a significant result (*p* < 0.001). Subsequently, for each determined factor, calculate the average value. This process generated factor scores ranging from 1 to 5, where higher scores reflect stronger agreement. Pearson’s correlation coefficient was utilized to examine the relationship between pairs of normally distributed continuous variables. All statistical significance tests were two-tailed, with p-values below 0.05 considered statistically significant. Analyses were performed using IBM SPSS Statistics Version 26.0. The suitability of the factor analysis data was evaluated through the Kaiser-Meyer-Olkin (KMO) measure for testing the sufficiency of sampling and the Bartlett test for sphericity.

### Qualitative data analysis

This study employed semi-structured interviews with five participants, followed by verbatim transcription of audio recordings and systematic thematic analysis guided by the methodological framework proposed by Braun and Clarke^20^. The analytical process comprised six sequential stages: (1) Becoming familiar with the data through repeated reading of the transcripts; (2) Generating initial codes; (3) Identifying potential themes by grouping related codes; (4) Reviewing and refining the candidate themes; (5) Defining and naming the final themes; and (6) Writing the final report. To ensure coding reliability, the first author and second author independently performed the coding process, with discrepancies resolved through collaborative discussion until full consensus was achieved.

## The results of quantitative research

### Demographic characteristics

**Fig. 2:**
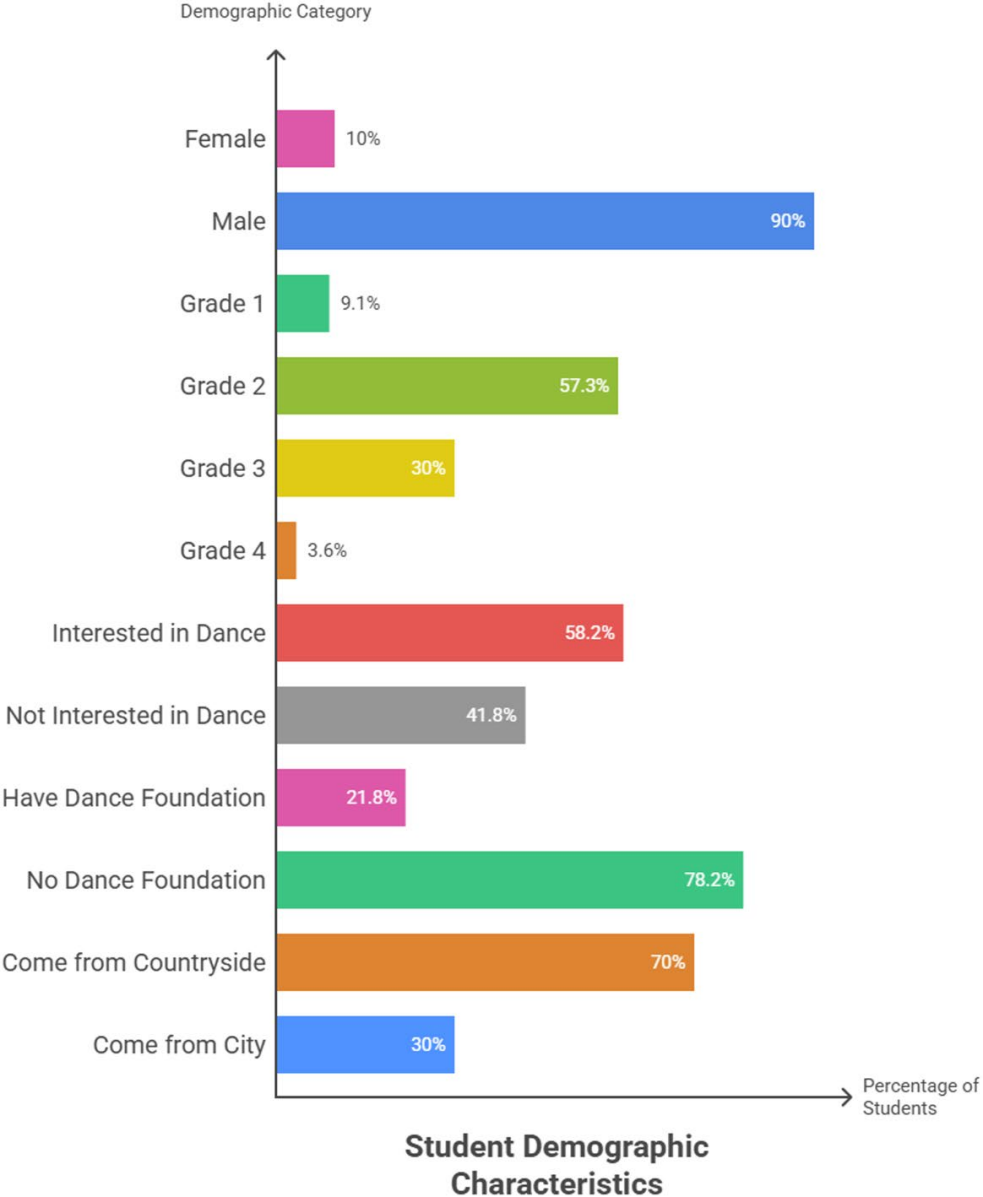
Student’s demographic characteristics.

Descriptive statistics are used to describe the demographics of the sample (from different socio-cultural backgrounds and achievement levels), including ‘Items, Number (N), Percent (%)’. The research samples are college students from Grade 1 to Grade 4. The basic information of 110 students who participated in this survey is elaborated in Fig. 2.

### Factor analysis

Respond to RQ2. Prior to conducting the Exploratory Factor Analysis (EFA), the data were assessed for suitability against key assumptions. It can be observed from Table 1 that the KMO value of the scale data was 0.926 > 0.6, and the Bartlett’s test of sphericity *P* = 0.000(< 0.05). This indicates that the data of questionnaire are extremely suitable for factor analysis. These results validate the MR-DAS as a structurally sound instrument for measuring its intended constructs.

**Table 1:** Adaptability test.

KMO and Bartlett’s Test		
Kaiser-Meyer-Olkin Measure of Sampling Adequacy.		0.926
Bartlett’s Test of Sphericity	Approx. Chi-Square	4360.966
	df	351
	Sig.	0.000

The results of exploratory factor analysis (EFA) are shown in Table 2. According to Table 2, the 29 items in the instrument are divided into 3 factors. Q9, and Q19 have no dimension, so we need to remove it.

**Table 2:** Exploratory factor analysis for the 29 questionnaire items.

Factors Derived from the Exploratory Factor Analysis			
Items	1	2	3
	Learning impact	MR teaching intention	Equipment requirements
15.If there are MR Dance games or teaching, I would be more willing to interact with my classmates.	1.014		
16.I think MR Dance games or teaching can play a better role in urging.	0.983		
17.Before taking MR dance course, it is very necessary for a teacher to guide.	0.951		
18.Before taking MR dance course, teacher’s guidance is needed.	0.872		
14.If there are MR Dance games or teaching, I would be more willing to interact with the teacher	0.851		
12.I think MR Technique is helpful for my dance studies.	0.791		
13.If there are MR Dance games or teaching, I would be more willing to learn.	0.755		
11. If I learn to dance online with a headset, I only need to find a teacher offline to correct my mistakes, which will not only save time but also get good results. I think MR dance will be a great teaching mode.	0.638		
10.If dancing with a headset allows me to complete my dance practice at home, I would prefer MR Dance teaching.	0.568		
26.My biggest interest in MR Dance teaching lies in its practicality.		0.961	
29.My biggest interest in MR Dance teaching lies in its interactive behavior.		0.917	
28.My biggest interest in MR Dance teaching lies in its learning atmosphere.		0.911	
23.I am not familiar with MR Dance teaching.		0.897	
27.My biggest interest in MR Dance instruction lies in its ease of use.		0.869	
24.MR Dance teaching is a new way of learning for me.		0.841	
25.I think MR Dance teaching can completely replace the online + offline blended teaching model.		0.759	
20.No instructor is required before taking MR dance course.		0.626	
22.I know a lot about the MR Dance teaching.		0.527	
21.I know Mixed Reality technology combined with dance teaching very well.		0.513	
3.I know a lot about MR(Mixed Reality) technique.			1.027
7.I don’t think it’s safe to dance with headsets on.			1.000
5.I know about the VR device Oculus Quest 2/3.			0.929
4.I know VR techniques is already being used in education.			0.855
2.I know a lot about the VR(Virtual Reality)technique			0.828
1.I know a lot about the AR(Augmented Reality)technique.			0.820
6.I am willing to wear a headset while dancing.			0.721
8.If I dance with a headset, MR dance can further reduce the number of hours of offline learning, I will be more willing to choose this kind of teaching.			0.617
9.If I dance with a headset that can make my dance moves more in place, I would prefer to choose MR Dance teaching.			
19.Before taking MR dance course, a teacher’s guidance is only needed sometimes.			

Values express loadings.

Principal Component Analysis extracted three components with eigenvalues greater than 1, which collectively accounted for 80.525% of the total variance before rotation; More details are summarized in the following Table 3. Based on the factor division and the meaning of the items, the author divides the factor dimensions into (a) Learning impact (b) MR teaching intention (c) Equipment requirements.

**Table 3:** Explanation of total variance.

Component	Initial Eigenvalues			Extraction Sums of Squared Loading			Rotation Sums of Squared Loading		
	Total	% of Variance	Cumulative %	Total	% of Variance	Cumulative %	Total	% of Variance	Cumulative %
1	20.087	69.267	69.267	20.087	69.267	69.267	7.959	27.446	27.446
2	1.796	6.192	75.459	1.796	6.192	75.459	7.88	27.174	54.62
3	1.469	5.066	80.525	1.469	5.066	80.525	7.512	25.905	80.525
4	0.969	3.343	83.868						
5	0.799	2.755	86.622						
6	0.455	1.569	88.191						
7	0.436	1.505	89.696						
8	0.376	1.296	90.993						
9	0.303	1.045	92.038						
10	0.283	0.975	93.013						
11	0.247	0.852	93.865						
12	0.238	0.82	94.685						
13	0.188	0.648	95.333						
14	0.173	0.597	95.93						
15	0.166	0.571	96.501						
16	0.137	0.473	96.975						
17	0.13	0.449	97.423						
18	0.11	0.379	97.803						
19	0.101	0.348	98.151						
20	0.1	0.346	98.497						
21	0.082	0.283	98.781						
22	0.07	0.24	99.021						
23	0.063	0.219	99.24						
24	0.06	0.205	99.445						
25	0.046	0.158	99.603						
26	0.038	0.133	99.736						
27	0.033	0.114	99.85						
28	0.028	0.098	99.948						
29	0.015	0.052	100						

Extraction Method: Principal Component Analysis.

### Reliability analysis

As for RQ1, it can be observed from Table 4 that MR DAS exhibits strong internal consistency, and the reliability test results are extremely satisfactory. According to the raw coefficient alpha and the Spearman–Brown coefficient, the questionnaire developed excellent reliability. In particular, all raw coefficients alpha values and Spearman–Brown coefficients were > 0.90. The raw coefficient alpha for the overall instrument was 0.983 and the Spearman-Brown coefficient was 0.929. The raw coefficient alpha values for the three factors that emerged from the factor analysis ranged from 0.965 to 0.973.

**Table 4:** Reliability statistics for the questionnaire.

Items	Factors Derived from the Exploratory Factor Analysis			Overall Instrument
	1	2	3	
	Learning impact	MR teaching intention	Equipment requirements	
Raw coefficient alpha	0.971	0.965	0.973	0.983
Spearman-Brown coefficient	0.925	0.913	0.962	0.929
Part 1	0.966	0.943	0.944	0.977
Part 2	0.948	0.949	0.955	0.966

### Descriptive statistics

Descriptive statistics for the 27 items and the three factors are presented in Table 5. Mean total scores for the three factors were above the mid-point value (= 3) of the scale, indicating Factor 1 learning impact (mean = 3.67), Factor 2 MR teaching intention (mean = 3.76), Factor 3 Equipment requirements (mean = 3.51). Regarding other aspects of the questionnaire, the students preferred to choose MR dance teaching as a new learning form (mean = 3.81), to replace blended teaching (mean = 3.78), students were focus on practicality (mean = 3.78), ease of use (mean = 3.81), learning atmosphere(mean = 3.80)and interactive behavior (mean = 3.87), although they are not familiar with MR dance teaching (mean = 3.76).

Respond to RQ4, the empirical findings of MR-DAS validate that optimizing future dance courses should prioritize four interconnected dimensions: practicality (as reflected by the average value of high factor 2: 3.64–3.87), ease of use (as evidenced by the load of device requirement factors: 0.582–0.804), a collaborative learning environment (as demonstrated by learning impact Q14–15, with an average score > 3.58), and technology-enhanced interaction (particularly Q 29, with the highest average score of 3.87). By engaging MR to dance teaching effectiveness, these elements collectively address the learning needs of college students in dance education.

Therefore, these data results findings suggest that students not only acknowledge the importance of teacher guidance but also demonstrate openness to adopting the MR teaching model as a primary instructional approach. In other words, students place strong value on the MR technology’s capabilities in ensuring teaching quality, providing real-time feedback, and enabling multi-dimensional observation. Meanwhile, human teachers are more focused on fostering creativity, solving personalized needs, guiding teaching, and ensuring student safety—functions that offer higher-level pedagogical support in these specific areas.

**Table 5:** Descriptive statistics for the 27 items and the three factors.

Items	Mean	SD	Median	Min	Max
1.I know a lot about the AR(Augmented Reality)technique.	3.44	0.873	3.42	1	5
2.I know a lot about the VR(Virtual Reality)technique.	3.48	0.865	3.46	1	5
3.I know a lot about MR(Mixed Reality) technique.	3.45	0.884	3.42	1	5
4.I know VR techniques is already being used in education.	3.52	0.843	3.48	1	5
5.I know about the VR device Oculus Quest 2/3	3.50	0.906	3.49	1	5
6.I am willing to wear a headset while dancing.	3.52	0.896	3.51	1	5
7.I don’t think it’s safe to dance with headsets on.	3.58	0.817	3.54	2	5
8.If I dance with a headset, MR dance can further reduce the number of hours of offline learning, I will be more willing to choose this kind of teaching.	3.59	0.827	3.56	2	5
10.If dancing with a headset allows me to complete my dance practice at home, I would prefer MR Dance teaching.	3.64	0.843	3.6	2	5
11. If I learn to dance online with a headset, I only need to find a teacher offline to correct my mistakes, which will not only save time but also get good results. I think MR dance will be a great teaching mode.	3.65	0.797	3.61	2	5
12.I think MR Technique is helpful for my dance studies.	3.64	0.810	3.59	2	5
13.If there are MR Dance games or teaching, I would be more willing to learn.	3.63	0.800	3.58	2	5
14.If there are MR Dance games or teaching, I would be more willing to interact with the teacher.	3.58	0.794	3.55	2	5
15.If there are MR Dance games or teaching, I would be more willing to interact with my classmates.	3.69	0.798	3.66	2	5
16.I think MR Dance games or teaching can play a better role in urging	3.69	0.798	3.66	2	5
17.Before taking MR dance course, it is very necessary for a teacher to guide.	3.74	0.809	3.71	2	5
18.Before taking MR dance course, teacher’s guidance is needed	3.73	0.834	3.7	2	5
20.No instructor is required before taking MR dance course	3.65	0.913	3.64	1	5
21.I know Mixed Reality technology combined with dance teaching very well	3.64	0.885	3.63	1	5
22.I know a lot about MR Dance teaching	3.65	0.861	3.64	1	5
23.I am not familiar with MR Dance teaching	3.76	0.812	3.74	2	5
24.MR Dance teaching is a new way of learning for me	3.81	0.840	3.8	2	5
25.I think MR Dance teaching can completely replace the online + offline blended teaching model	3.78	0.882	3.78	2	5
26.My biggest interest in MR Dance teaching lies in its practicality	3.78	0.828	3.77	2	5
27.My biggest interest in MR Dance instruction lies in its ease of use	3.81	0.818	3.78	2	5
28.My biggest interest in MR Dance teaching lies in its learning atmosphere	3.80	0.810	3.8	1	5
29.My biggest interest in MR Dance teaching lies in its interactive behavior	3.87	0.779	3.87	2	5

SD: Standard deviation.

### Correlation analysis and result analysis

The author chose Pearson correlation analysis to examine the relationship between Factor 1 Learning impact, Factor 2 MR teaching intention, Factor 3 Equipment requirements and the overall intention. The results are shown in Table 6 revealed that the overall intention was firmly and positively correlated with Factor 1 Learning impact (*r* = 0.926, *p* < 0.01), Factor 2 MR teaching intention (*r* = 0.932, *p* < 0.01), and Factor 3 Equipment requirements(*r* = 0.933, *p* < 0.01). It means there is a positive correlation between those three variables. 

**Table 6:** Pearson correlation analysis result above the factors.

	Learning impact	MR teaching intention	Equipment requirements	Overall satisfaction
Learning impact	1			
MR teaching intention	0.780**	1		
Equipment requirements	0.812**	0.804**	1	
Overall intention	0.926**	0.932**	0.933**	1

** Correlation is significant at the 0.01 level (2-tailed).

Factor 1 Learning impact and Factor 2 MR teaching intention show a significant positive correlation, with a correlation coefficient of 0.780; Factor 1 Learning impact and Factor 3 Equipment requirements show a significant positive correlation, with a correlation coefficient of 0.812; Factor 2 MR teaching intention and Factor 3 Equipment requirements show a significant positive correlation, with a correlation coefficient of 0.804.

The correlation between Factor 2 MR teaching intention and Factor 1 Learning impact is the lowest in this study, which means that in this study, the correlation between students’ learning intention to integrate MR technology into teaching and whether their own learning is affected is only 0.780. The correlation between Factor 1 Learning impact and Factor 3 Equipment requirements ranks second, which proves that the relationship between whether students are affected by learning and the equipment they use is 0.812. The correlation between Factor 2 MR teaching intention and Factor 3 Equipment requirements is the highest in this study (0.804), which shows that the teaching technology used by teachers has a great relationship with what equipment students use in class.

## The results of qualitative research

Thematic analysis of the interview records revealed several key themes regarding students’ acceptance of MR dance teaching. We have listed these themes along with direct quotes from the participants in Table 7. Through semi-structured interviews with 7 respondents (with an average duration of 28 min), the study revealed that students’ acceptance of Mixed Reality (MR) Dance teaching exhibits a ternary structure characterized by “high expected utility,” “moderate technical cognition,” and “low safety perception.” This structure aligns closely with the Correlation Analysis. Furthermore, equipment requirements demonstrate a strong correlation with “Learning impact” and “MR teaching intention” (*r* = 0.780–0.812, *p* < 0.01), which is corroborated by the high factor loading of “Equipment requirements” (0.692–0.838). To further validate the quantitative analysis findings, feedback from telephone interviews with students was collected and synthesized as follows:

### The symbolization of technological cognition and the knowledge gap

In the research, the author found that some respondents would have a confusion of concepts. Feedback from a freshman said that MR was an upgraded version of VR, she was unable to see the real environment around, and she equated MR with “advanced VR”. This contrasts with the average understanding of technical knowledge (3.44–3.52) from Q1 to Q4 in this study. This feedback confirmed the phenomenon of Symbolic Technology Awareness^24^.

The author realized that part of respondents indicated a willingness to wear headsets while dancing, the same as the result of Q6, primarily due to safety concerns among participants. As reflected in Q 7 (Mean = 3.58), some students commented, “During rotations, the headsets tend to shift, obstructing the field of vision and raising concerns about potential collisions with furniture.” This suggests that in the context of technological application, lighter and more secure equipment can enhance user comfort and safety perception. Consequently, the findings related to technological cognition and device usability, which correspond to factors 1 and 2 in the scale. This result further validates the reliability and credibility of the measurement tool.

### The anticipated impact of MR enhances the willingness to learn and accept

 According to Table 4 in Sect. Reliability analysis of this article, it can be observed that the reliability analysis results for the three factors of this scale are satisfactory (Cronbach’s α = 0.965–0.973). Additionally, Factor 1 (learning influence) exhibits a strong correlation with Factor 2 (teaching willingness) (*r* = 0.780). To further validate the findings of RQ 4, based on feedback from five representative students and in conjunction with the results of the scale analysis, the author concludes that students’ adoption decisions are significantly influenced by the three key factors: “efficiency,” “accuracy,” and “space liberation,” as illustrated in Table 7.

**Table 7:** MR-Enhanced dance pedagogy: triadic utility dimensions driving learning Adoption.

Utility Dimension	Qualitative Evidence	Scale Association
Time compression	“MR can cut down on the time I spend fixing errors offline, which makes it really great for repetitive training during practice.” (Student A)	Q11 (Load = 0.638)
Dance moves optimization	“I think the timely feedback function of overlapping virtual and real movements can make the dance visual, which is more direct than the teacher’s verbal guidance” (Student B)	Q12 (Load = 0.791)
Spatial emancipation	“If one could practice dancing at home, it would surely be a revolutionary breakthrough. However, some problems existing in multi-person collaborative work need to be solved, such as movement delay and unclear imaging. (Student C)	Q18 (Load = 0.617)
Teacher Role Transformation Catalyst	“We think teachers should focus on cultural interpretation and creative inspiration. Repetitive training can be completely entrusted to MR.” (Student D)	Q17-18 (Mean > 3.73)
Gamified Progression Engine	“During the learning-through-play process, the system’s evaluation metrics, ranking features, and virtual reward mechanisms significantly enhanced my engagement. This experience was akin to the “MR version” of the “Just Dance” game, where players unlock progressively more challenging levels upon successfully completing prior ones. Such interactive design elements greatly motivated me and transformed what might otherwise have been a monotonous dance practice into an enjoyable and stimulating activity. This is very attractive for students like me with no dance foundation.”. (Student E)	Q13-16 (load > 0.721)

### Cognitive reconstruction of the teacher role and gamification expectations

According to the results of factor analysis and telephone interviews, it is known that students’ cognitive reconstruction and expectations of dance gamification are also critical factors for RQ4. 60% of the students support the role transformation of teachers. Student D stated, “Teachers should focus on cultural interpretation and creative inspiration. Repetitive training can be entirely entrusted to MR.” Meanwhile, in the quantitative analysis, Q17-18 (Mean > 3.73) also supplemented this point. It is worth noting that a student E with no foundation expressed that she was looking forward to MR Game teaching. If she could learn and play through games at the same time, set up a ranking system for each level and a virtual reward mechanism, just like the MR Version of the “Just Dance” game, unlocking the level games after passing each level would stimulate the motivation to keep practicing. In quantitative analysis, Q13-16 (load > 0.721) also precisely indicates that gamification design can enhance the stickiness of scale research.

Based on the data in Table 7, we derived a new conclusion. Student D definitely articulated the expectation that teachers should focus on fostering creativity in the classroom and expanding students’ cognitive horizons, such as by integrating MR technology into the learning environment. In other words, there is a growing hope for a transformation in the role of teachers. Similarly, Student E’s expectations regarding MR-based instruction suggest that this technology may assume some of the responsibilities traditionally associated with foundational teaching. These perspectives offer direct insight into the evolving role of educators in the future.

### Concerns regarding the security of the technology have emerged as a critical barrier to its widespread adoption

Some students have expressed concerns regarding the physical risks associated with technology use. For instance, like Q7, when discussing the use of head-mounted displays, a potential collision caused by motion-capture blind spots was raised. One student stated, “I am concerned that during large jumps or vigorous movements in a dance, if the system warning is delayed by 0.5 seconds, I might sustain an injury.” Another remarked, “If the application of technology can alleviate my cognitive load, I would be willing to embrace it. After all, no one prefers rote memorization of dance movements.” This constitutes an empirical response to Sweller’s cognitive load theory proposed in 1988^25^. Sweller posted that cognitive load related to meaningful learning and problem-solving could be effectively managed through instructional design and presentation using Mixed Reality (MR) technology. Consequently, the concept of optimizing learning aligns closely with Sweller’s cognitive load theory, further validating the rationale of the scale employed in this study. Therefore, concerns regarding cognitive symbols, physical risks, and technical security remain pivotal factors in determining the extent of its widespread adoption.

In this session, the results of the qualitative analysis uncover the core factors influencing the implementation of MR Dance teaching. Multidimensional tension of Technology Acceptance and Consensus on Educational utility, it addresses RQ 4 regarding the key areas that should be emphasized in learners’ needs within the context of MR dance education moving forward. This study provides a valuable research foundation and reference for educators interested in further exploring the application of emerging technology, specifically MR, in dance instruction. Furthermore, the feedback obtained from students offers empirical support for technological advancement and educational integration, aligning with the sustainable development goal of SDG4 (Quality Education).

## Discussion

The findings of this study confirm the psychometric validity of the 27-item Mixed Reality Dance Attitude Scale (MR-DAS), providing robust evidence of its clear three-factor structure, high internal consistency, and satisfactory construct validity, thereby establishing it as a reliable and valid instrument for its intended application.

### Positioning the study’s contribution and instrument validation

With the increasing advancement and convenience of science and technology, it is highly significant to investigate the online teaching of art and tailor it to various groups of people in order to mitigate the impact of special circumstances, such as epidemics, on teaching effectiveness. To the best of our knowledge, this represents the first study to develop an instrument for assessing intentions regarding an MR-enhanced dance course. This survey explores the perspectives of Chinese college students on integrating the emerging technology of Mixed Reality (MR) into dance education. The instrument underwent rigorous testing and demonstrated satisfactory psychometric properties, establishing itself as a reliable and valid tool for future research in this domain. Specifically, the total variance explained before rotation was explained 80.525%, indicating its statistical appropriateness and robustness, including Learning impact, MR teaching intention and Equipment requirements.

Regarding the dimension of Learning Impact, we contend that the selection of any technological intervention and its influence on students’ learning outcomes are of paramount importance, as evidenced by numerous studies^26,27^.

Since the author of this article is working on a creative dance teaching module, the preliminary research on the use of MR technology in dance teaching is crucial. This research focuses on students’ intention to participate in MR technology-based dance classes, which directly impacts the smooth implementation of subsequent MR dance courses. Equipment requirements play a significant role in enhancing students’ learning intention for MR dance courses. The instrument was rigorously tested and demonstrated satisfactory psychometric performance, making it a valid tool for future research in this area.

### Interpreting the Three-Factor structure

One of the primary findings of this study is that students exhibit a strong intention toward MR teaching (Q25 mean = 3.76), with the highest correlation observed in relation to equipment requirements. Despite their limited familiarity with MR dance teaching (factor loading = 3.76), several key factors enhance their willingness to engage in MR dance learning, including practicality, ease of use, learning atmosphere, and interactive behavior offered by MR dance classes. Furthermore, to respond to RQ4, based on the results of Q25, it is evident that students demonstrate a strong level of acceptance toward the MR teaching approach and maintain an open attitude toward the proposed “alternative hybrid model.” However, the author argues that this should not be interpreted merely as a sign of “replacing human teachers.” When considering students’ acknowledgment of the importance of teacher guidance (as reflected in Q17 and Q18), along with interview suggestions emphasizing teachers’ roles in providing “cultural interpretation” and fostering “creative inspiration,” this study supports the perspective that MR technology facilitates a ‘role reconfiguration’ rather than a ‘role replacement’ of educators. It is anticipated that MR will assume responsibilities related to standardized instruction, repetitive tasks, and immediate feedback. Through technological empowerment, teachers can redirect their efforts toward addressing diverse student needs and engaging in more creative and impactful teaching practices.

Recent research shows that, while motion capture has enriched dance documentation, its integration with MR interfaces for pedagogical innovation lacks standardized frameworks^28,29^. The absence of validated instruments impedes cross-study comparability. Some scholars have noted that the integration of MR technology into dance education systems lacks standardized criteria for evaluating dancers^27^. This article proposes the application of Gardner’s multiple intelligence theory as a robust theoretical foundation for developing an assessment tool, thereby addressing the existing gap in this domain. The following section presents the response to RQ3.

### Student perceptions of MR as a catalyst for multiple intelligence: empirical evidence and pedagogical implications

Based on the Exploratory Factor Analysis (EFA) results and the application of Gardner’s Multiple Intelligence (MI) framework to the MR-DAS data, there is compelling empirical evidence indicating that students perceive MR dance education as fostering multiple intelligence. Table 8 is a structured analysis:

**Table 8:** The evidence linking MR-DAS content to gardner’s MI Domains.

MI Domain	Items	Supporting MR-DAS Factors	Key Evidence
Logical-Mathematical (Technology ease/perception)	3,4, 5,6	Factor 3: Equipment Requirements (MR knowledge, device willingness)	High loading (0.721–1.027) positive means (3.45–3.52) indicate students logically appraise the equipment willingness of MR.
Body-Kinesthetic (Dance ability/intention)	11,12 13,16	Factor 1: Learning Impact (MR improves dance learning, motivation, self-correction)	Strong loading (0.638–0.983) high means (3.63–3.69) show perceived enhancement of physical learning.
Musical (Rhythm/skill efficiency)	26,27	Factor 2: MR Teaching Intention (interest in practicality/ease)	Loading (0.917-0.961) highest means (3.78–3.81) confirm intent to adopt MR for skill/rhythm mastery.
Interpersonal (Virtual collaboration)	14,15	Factor 1: Learning Impact (willingness to interact with teachers/peers)	Strong loading (0.851-1.014)) high means (3.58–3.69) demonstrate how MR facilitates social interaction.

The empirical evidence presented in this study suggests that MR dance education is perceived by learners as a viable catalyst for fostering Gardner’s multiple intelligence (MI). The exploratory factor analysis (EFA) results demonstrated that the three robust dimensions of the Mixed Reality Dance Acceptance Scale (MR-DAS) align closely with four intelligence within the MI framework.

Body-Kinesthetic(BK) intelligence was substantiated by strong factor loading (ranging from 0.638 to 0.983) on items related to learning impact, such as the role of MR in enhancing dance skill acquisition and self-correction capabilities. Additionally, this was reinforced by strong participant agreement, with mean scores consistently at or above 3.63.

Interpersonal(INR) intelligence was evidenced by items indicating enhanced social interaction with peers and instructors (factor loading: 0.851-1.014); mean: 3.58–3.69), It has been verified that MR technology with the potential development of their Interpersonal intelligence.

Overall, the MR-DAS analysis suggests that students perceived MR-based dance teaching as a tool with the potential to facilitate the development of multiple intelligence. This suggests that students associated their understanding of MR technology with the potential development of their Logical-Mathematical intelligence.

Musical (MUL) intelligence gains were evidenced by high interest in MR’s practicality for rhythm/spatial mastery (loading: 0.917-0.961; mean: 3.78–3.81). Through the analysis we understand that students perceived MR technology as a tool with the potential to facilitate the development of musical intelligence.

Logical-Mathematical(LM) intelligence was reflected in respondents’ systematic appraisal of MR usability (loading: 0.721-1.027; mean: 3.45–3.52), This confirms that students, through their understanding of MR Technology knowledge, equipment familiarity and willingness to use it, realize that its technical feasibility as potentially fostering their Logical-Mathematical intelligence.

Overall, students perceived that MR-based dance teaching has the potential to support the development of MI (including BK, INR, MUL and LM) development of students.

### Bridging innovation and evidence

Notably, in contrast to recent empirical studies that have reported non-significant effects of perceived ease of use on behavioral intentions within technology-mediated learning environments^30^, our findings highlight a significant divergence. Despite participants’ relatively limited prior experience with MR dance pedagogy, they demonstrated elevated expectations regarding its perceived ease of use. Although the students involved in this study lacked prior experience with MR dance instruction, they demonstrated notably higher expectations regarding its perceived ease of use. This lays a sustainable development foundation for scholars who wish to further explore the integration of MR technology into dance education in the future.

There is substantial evidence indicating that traditional art forms have undergone transformation with the integration of modern technologies. Specifically, the emerging MR technology has introduced novel possibilities for innovation in artistic expression^31^. The digital era, characterized by Virtual Reality (VR), Augmented Reality (AR), and Mixed Reality (MR), is redefining conventional teaching methodologies, particularly within the domain of experiential learning^32^. The immersive and interactive attributes of these advanced technologies offer learners unprecedented opportunities while fostering rich and dynamic educational experiences^33^. However, the integration of these technologies into educational settings is not without significant challenges, encompassing technological constraints, pedagogical concerns, and potential health implications that require systematic and methodological attention^34^. The utilization of emerging technologies in teaching can facilitate students’ conceptual learning within simulated environments, thereby fostering deeper comprehension and enhancing knowledge retention^35^. As technology continues to advance, the synergy between emerging technologies and experiential learning is anticipated to transform the educational landscape for future generations^36^.

Some scholars have highlighted that when considering the impact of emerging technologies on learning, their capacity to bridge theory and practice becomes particularly noteworthy^32^, the learning impact and the intention to use Mixed Reality (MR) technology, with a correlation coefficient of 0.780 (*p* < 0.01). Moreover, the relationship between the learning impact and the overall intention to adopt this MR-based dance course is greatly significant, as indicated by a correlation coefficient of 0.926 (*p* < 0.01). These results suggest the participants hold a favorable attitude toward the application of the emerging MR technology in educational contexts.

Therefore, the MR-DAS developed in this study not only reflects students’ demand for technological integration in education, but also establishes a foundation for understanding the future human-machine collaboration (Human-MR Collaboration) dance teaching model. The author posits that the successful implementation of MR technology in educational contexts does not reside in replacing human instructors, but instead in leveraging the advantages of such technology to redefine the role and value of educators.

### Theoretical and practical significance

This study developed the MR-DAS instrument based on Gardner’s theory of multiple intelligence, establishing a three-factor measurement framework with strong reliability and validity. The scale not only addresses the lack of theoretical instruments for quantitatively assessing technology acceptance in dance education but also provides a foundational framework for understanding the multidimensional nature of students’ acceptance of mixed reality (MR) technologies.

Meanwhile, MR-DAS serves as a valuable instrument for dance educators engaged in empirical research. Dance instructors can conduct diagnostic assessments of students’ readiness and concerns prior to incorporating MR technology into instructional settings, enabling the development of more targeted and pedagogically sound curricula. This also establishes a foundational framework for scholars pursuing “student-centered” educational inquiries. For researchers, this study offers a standardized metric to benchmark and compare the effectiveness of MR integration across diverse contexts, thus advancing evidence-based practices in emerging technology-enhanced education.

## Limitation

There are also certain limitations in this study. Specifically, three items were found to simultaneously load on two dimensions during the exploratory factor analysis (EFA), which necessitates targeted revisions. However, due to time constraints, the content of these three items was not revised further, the questionnaires were not redistributed, and additional data collection was not conducted. Although these items were retained in this initial validation phase to ensure adequate content coverage, future studies should:

### Enhance discriminant validity through semantic refinement of item wording

In the factor analysis, it was found that Q9 and Q19 had no factors (refer to Table 2), indicating that the wording of these two items might be ambiguous. However, the author considered that the content coverage of these two items was sufficient, scholars would conduct deeper research and revisions in the future, to further enhance the reliability and validity of the study through semantic refinement of the project wording. Additionally, for other items, the author suggests that to ensure the instrument accurately assesses students’ motivation and sustainable development skills related to MR dance education, the content should consistently focus on a student-centered foundation.

### Motivation insights for Future-Ready learning

Based on the author’s extensive experience in teaching dance courses, several key factors can significantly influence students’ learning motivation. These include teacher-student interaction, instructional guidance, the degree of technical integration, and the appropriateness of teaching methods. Such factors not only affect the boundaries of educational dimensions but are also critical for promoting sustainable education and fostering innovative practices. Future research should focus on integrating students’ learning contexts to enable educators to design more personalized teaching strategies, effectively leverage emerging technologies, and determine optimal approaches for their research objectives. The future direction of educational research should emphasize scale development, such as group collaboration, adaptive learning systems, and personalized teaching plans. This adaptability is essential for cultivating relevant skills for sustainable development in higher education, ensuring that student-centered learning remains effective, and addressing evolving trends and challenges in higher education. Ultimately, this research will provide actionable insights into the digital transformation of dance education, guiding it toward a more practical and contextually relevant learning experience while preparing students for future demands.

## Conclusion

To advance the Sustainable Development of higher education, universities may then implement such rigorously tested models through technology-enhanced teaching and personalized training courses^17^. The MR-DAS instrument demonstrated satisfactory psychometric properties in this study, supported by its theoretically grounded structure, which maps Gardner’s Multiple Intelligence onto MR-specific constructs. This instrument offers researchers a validated framework to analyze how domain-specific intelligence mediate Mixed Reality (MR) adoption, thereby enabling targeted optimization of dance pedagogy across technological, embodied, aesthetic, and social dimensions.

The development of the MR-DAS scale involved conducting an intention survey to evaluate the teaching effectiveness of MR technology in dance education and established a standardized framework. This instrument provides a foundation for assessing and refining dance educational practices, thereby contributing to the broader goals of SDG 4 “Ensuring Inclusive and Equitable Quality Education,” and is applicable to students globally. As an intention survey, MR-DAS enables educators to continuously refine immersive teaching models, thereby accelerating the sustainable integration of emerging technologies into education. Ultimately, it will serve as an empirical foundation for realizing the vision of SDG 4, “Lifelong Art Learning for All,” within the context of educational digital transformation.

This study offers an empirical foundation for educators to assess and develop more effective and student-centered MR-based dance pedagogical strategies. The study highlights students’ strong expectations for MR technology to enhance both learning efficiency and experiential quality, while also emphasizing the irreplaceable role of human instructors in critical areas such as creative guidance, cultural interpretation, and safety assurance. Looking ahead, the practical application of MR-DAS can contribute to the achievement of Sustainable Development Goal 4 (SDG 4).

At the level of teaching practice, this scale enables educators to accurately identify students’ diverse learning needs, support instruction tailored to individual aptitudes, and facilitate the development of more personalized and engaging dance curricula.

At the level of digital transformation, this study provides insights into the future direction of dance education: It is essential to systematically explore the synergistic potential between mixed reality (MR) technology and human instructors, with emphasis on leveraging the complementary strengths inherent in human-machine interaction, thereby fostering high-quality, personalized, and sustainable dance learning experiences through a collaborative human-machine teaching framework.

Ultimately, applying MR-DAS within a broader framework of cross-cultural comparative studies will contribute to uncovering pathways toward achieving equitable, high-quality art education on a global scale, thereby enabling this tool to make a tangible contribution to the realization of the Sustainable Development Goals^23^.

## Data Availability

The datasets acquired during the current study are available from the corresponding author upon reasonable request.
